# Vacuolar Protein Sorting Receptor in *Giardia lamblia*

**DOI:** 10.1371/journal.pone.0043712

**Published:** 2012-08-20

**Authors:** Maria R. Rivero, Silvana L. Miras, Constanza Feliziani, Nahuel Zamponi, Rodrigo Quiroga, Stanley F. Hayes, Andrea S. Rópolo, Maria C. Touz

**Affiliations:** 1 Instituto de Investigación Médica Mercedes y Martín Ferreyra, Universidad Nacional de Córdoba, Córdoba, Córdoba, Argentina; 2 Departamento de Química Biológica, Facultad de Ciencias Químicas, Universidad Nacional de Córdoba, Córdoba, Córdoba, Argentina; 3 Rocky Mountain Laboratory, NIAID, National Institutes of Health, Hamilton, Montana, United States of America; Institut Curie, France

## Abstract

In *Giardia*, lysosome-like peripheral vacuoles (PVs) need to specifically coordinate their endosomal and lysosomal functions to be able to successfully perform endocytosis, protein degradation and protein delivery, but how cargo, ligands and molecular components generate specific routes to the PVs remains poorly understood. Recently, we found that delivering membrane Cathepsin C and the soluble acid phosphatase (AcPh) to the PVs is adaptin (AP1)-dependent. However, the receptor that links AcPh and AP1 was never described. We have studied protein-binding to AcPh by using H6-tagged AcPh, and found that a membrane protein interacted with AcPh. This protein, named GlVps (for *Giardia lamblia* Vacuolar protein sorting), mainly localized to the ER-nuclear envelope and in some PVs, probably functioning as the sorting receptor for AcPh. The tyrosine-binding motif found in the C-terminal cytoplasmic tail domain of GlVps was essential for its exit from the endoplasmic reticulum and transport to the vacuoles, with this motif being necessary for the interaction with the medium subunit of AP1. Thus, the mechanism by which soluble proteins, such as AcPh, reach the peripheral vacuoles in *Giardia* appears to be very similar to the mechanism of lysosomal protein-sorting in more evolved eukaryotic cells.

## Introduction

Trafficking of newly synthesized lysosomal soluble enzymes from the trans-Golgi network (TGN) to lysosomes in mammalian cells occurs indirectly, via the plasma membrane followed by endocytosis, or directly, via the endosomal system. In these cells, the mannose 6-phospate receptors (MPRs) bind with high affinity to phosphomannosyl residues attached to the ligand proteins, while their cytoplasmic tails contain motifs capable of recognizing components of the clathrin-coated vesicles that are directed to the endosome/lysosome pathway. In the endosome, the receptor-ligand complexes are dissociated and the receptor is recycled to a late Golgi compartment, while the hydrolase ends up on the lysosome (reviewed by [1], [2]). In a similar way, sorting of soluble vacuolar hydrolases (e.g. carboxypeptidase Y, CPY) in yeast depends on the VPS10p (vacuolar protein sorting 10 protein) [3], although the sorting is dependent, not on oligosaccharide residues [4], but on sequences in the vacuolar proteins or a structural determinant that might be recognized directly by the receptor [5]. While in the TGN, the MPRs and Vps10p bind soluble hydrolases, their cytoplasmic tail packages them into clathrin-coated vesicles, aided by adaptor proteins. Heterotetrameric adaptor-protein (AP) complexes or the GGA (Golgi-localized, γ-ear-containing, Arf-binding family of proteins) monomeric adaptor proteins recognize a tyrosine (Y)-based signal (YXXΦ) with a large hydrophobic residue (Φ) or a dileucine motif ([DE]XXXL[LI]) on cytosolic domains of these membrane receptors, respectively [2]. Proper adaptor binding will result in delivery of hydrolases to the late endosomes [2], [6].

Lysosomal protein trafficking in the early branching protist *Giardia lamblia* (syn. *Giardia duodenalis*, *G. intestinalis*) seems to be conserved, although some interesting particularities are present [7], [8]. First, this parasite lacks a defined Golgi apparatus for the sorting station [8], [9], [10] and the endosomal/lysosomal system is being represented by unique organelles defined as peripheral vacuoles (PVs) [11], [12], [13], [14]. While lysosomal membrane proteins follow the mammalian principles of binding to adaptor protein complexes (AP1 or AP2) and clathrin [13], [14] to be delivered to the PVs, the sorting and trafficking of soluble hydrolases remains unknown in this parasite. Previously, we observed that the soluble acid phosphatase (AcPh) of *G. lamblia* localized in the PVs and in the endoplasmic reticulum and was present in the nuclear envelope [13], in agreement with the enzyme cytochemical localization [11]. Also, we reported that depletion of the medium subunit of AP1 resulted in AcPh retention in the endoplasmic reticulum (ER), with this enzyme being mislocalized from the PVs [13]. These results indicated the presence of a membrane protein receptor in order for the AcPh to be successfully delivered to the PVs.

In this report, we show that a membrane protein named GlVps (for *Giardia lamblia* Vacuolar protein sorting), is localized to the ER-nuclear envelope and might serve as the sorting receptor for AcPh. GlVps subcellular localization depended on the presence of an YXXØ motif and on the medium subunit (µ1) of AP1. Because acid phosphatase activity decreased upon receptor down-regulation, we suggest that it might be involved in AcPh trafficking toward the PVs. These findings will advance our understanding of the molecular mechanism underlying soluble hydrolase sorting, opening new avenues for the comprehension of lysosomal protein-trafficking in parasites.

## Materials and Methods

### Antibodies and other reagents

Anti-HA and anti-V5 mAb were purchased from Sigma (St. Louis, MO). 9C9 mAb was employed to detect the ER-BiP protein [15]. Anti-µ2 mAb 2F5 was used for the µ2 subunit of AP2 [14]. 5C1 mAb was used to detect VSP1267 [16]. Alexa Fluor 488 and 555 was used for the primary antibody label (Zenon Tricolor Mouse IgG1 Labeling Kit, Molecular Probes, Invitrogen). Digitonin and Triton X-100 were also purchased from SIGMA.

### *Giardia* cell lines and vectors

Trophozoites of the isolate WB, clone 1267 [17] were cultured in TYI-S-33 medium supplemented with 10% adult bovine serum and 0.5 mg/ml bovine bile, as previously described [18]. These trophozoites were used as hosts for the expression of transgenic genes and as wild-type controls. **p**TubAcPh-V5/H_6_pac that carries the AcPh gene and the sequences encoding the V5 epitope plus six His at the C-terminus was used to stably express the AcPh-V5/H_6_ fusion protein [12]. The GlVps open reading frame was amplified from genomic DNA using the F1 (CATTGGGCCCCAGCACCCCGTTTTACTCGAA) and R1 (CATTCCCGGGGACGATGATTTGGTAGACCGT) primers and cloned into the plasmid **p**TubHAc-pac [12] to generate the **p**GlVps-HA vector. The mutant of GlVps lacking the lysosomal motif was amplified using the F1 and R2 (CATTCCCGGGGACCGTCCGGCCGAAAGCTACCAGGCA) primers and cloned into the **p**TubHAc-pac vector to generate the **p**GlVps_-YQII_-HA expression plasmid. Stable episomal transfection was performed as previously described [13], [19], [20], [21], [22]. All vectors contained a puromycin cassette under the control of the endogenous non-regulated *gdh* promoter for cell selection. Drug-resistant trophozoites were usually apparent by 7–10 days post-transfection. Trophozoites transfected with dsRNA-μa plasmid (for μ 1 knock-down) [13] were cotransfected with the **p**GlVps-HA vector, selected with puromycin, grown in complete medium and the μ 1 antisense induced with 10 µg/ml of tetracycline. For GlVps antisense, the first 1000 nt of the ORF was amplified using the ASf (CATTCCCGGGATGCAGCACCCCGTTTTACTCGAAA) and ASr (CATTCCATGGTGCAAGCAGCATTAGAGGACCGGAT) primers, restricted and ligated to the **p**TubHAc-pac in the opposite direction resulting in the antisense vector that was used for inhibition of GlVps expression. Trophozoites transfected with empty **p**TubHAc-pac plasmid was used as control.

### Immunoelectron Microscopy

*Giardia* trophozoites were rinsed twice with PBS and 0.1% growth medium, chilled, attached to Thermanox coverslips (Nunc, Naperville, IL) and reacted for 2 h in fixative containing 3 parts solution A (0.1 M lysine–HCl–NaPO_4_), 1 part solution B (8% paraformaldehyde, 21.3 mg of sodium periodate, and 100 μl of 25% glutaraldehyde), and an additional 0.1% glutaraldehyde. The coverslips were then rinsed in PBS and permeabilized with 0.05% saponin in PBS for 5 min at room temperature. For immunostaining, anti-V5 mAb diluted 1∶1000 in a 3% globulin-free bovine serum albumin (BSA)-PBS (Sigma) solution was added at room temperature for 1 h. After PBS-BSA washes, the 1.4 nm fluoronanogold anti-mouse immunoglobulin G Fab antibody (Nanoprobes, Yaphank, N.Y.), diluted 1∶30 in PBS-BSA with either 0.05% saponin, was added for 1 h at room temperature. The coverslips were washed five times in PBS and stored at 4°C in postfixative (2.5% glutaraldehyde, 4% paraformaldehyde) until they were used. The coverslips were washed in H_2_O and reacted for 4 min in the dark with a solution of HQ silver reagents (Nanoprobes) at an equal ratio of red-blue-white to enhance the signal. The coverslips were then washed three times in H_2_O and one time in 1% aqueous tannic acid for 5 min, followed by an H_2_O rinse. Next, the coverslips were reacted with a solution of reduced K_4_(FeCN)_6_ and 1% osmium tetroxide for 15 min, followed by two rinses in H_2_O. They were then subjected to a 5-min graded alcohol dehydration series of 50, 80, 95, and 100%, infiltrated with Spurr's resin, and polymerized at 60°C. The samples were then sectioned and examined using a Hitachi H7500 electron microscope equipped with a Hamamatsu digital camera (Advanced Microscopy Techniques Corp., Danvers, Mass.). The resulting images were digitally recorded.

### ELF97 Endogenous Phosphatase Substrate

ELF97 phosphatase substrate [2-(5-chloro-2-phosphoryloxyphenyl)-6-chloro-4-(3H)quinazolinome(CPPCQ)] (Molecular Probes; Eugene, OR) was used to test phosphatase activity following the protocol suggested by the company. Diverse concentrations of ELF97 and incubation times were tested; with the 20 uM concentration and 15 min incubation being the most appropriate conditions. The wild-type and transgenic trophozoites were incubated with ELF97 substrate in 110 mM acetate buffer (pH 5.5), containing 1.1 mM sodium nitrite (for acid phosphatase activity) or 10 mM potassium phosphate buffer (pH 7.0) (for alkaline phosphatase activity) [23], [24], [25], [26]. The fluorescence signal was analyzed and documented by conventional fluorescence microscopy, using a 364 nm Argon laser (Carl Zeiss Axiovert 35M) and captured with a silicon-intensified target camera (SIT-C2400; Hamamatsu Phototonics, Bridgewater, NJ). For quantitative fluorescent measurements of ELF 97, the Fiji image processing package (http://fiji.sc/wiki/index.php/Fiji) was used. Differences among groups were analyzed using the Mann–Whitney U test.

### Pull-down assay

AcPh-V5/H_6_ and wild-type trophozoites were grown, harvested and suspended in 1 ml of lysis buffer (50 mM NaH_2_PO_4_, 300 Mm NaCl, 10 mM imidazol, pH 8.0, 1% Triton X-100, and protease inhibitors) for 3 hours at 4°C. After mild sonication using a Branson sonifier 250 (Branson, CT) with an output control of 3 and a 50% duty cycle (sonication complex), the lysate was centrifuged at 7500 g, for 30 minutes at 4°C. Each supernatant was then mixed with 200 μl of Ni-agarose beads (QIAGEN, Valencia, CA) and incubated for 4 hours at 4°C. Beads were spun down at 700 g and washed four times with wash buffer (50 mM NaH_2_PO_4_, 300 mM NaCl, pH 8.0, 0.1% Triton X-100, and protease inhibitors). Bound proteins were eluted four times with 100 μl elution buffer (50 mM NaH_2_PO_4_, 300 mM NaCl, 250 mM imidazol, pH 8.0, 0.1% Triton X-100, and protease inhibitors). AcPh-V5/H_6_-bound proteins were analyzed by SDS-PAGE, stained with Coomassie G-250. The detected bands were cut out and submitted to the Research Technologies Branch for Protein Identification (NIAID, NIH) for LC-MS/MS analysis. After three independent experiments, three proteins that were associated with AcPh-V5/H_6_ were identified (Table S1).

### Immunoblot Analysis

Immunoblot assays were performed as previously reported [19]. Briefly, 10 µg of total proteins were incubated with sample buffer, boiled for 10 min, and separated in 10% Bis-Tris gels. Samples were transferred to nitrocellulose membranes, blocked with 5% skimmed milk and 0.1% Tween 20 in TBS, and then incubated with primary antibody diluted in the same buffer. After washing and incubation with an enzyme-conjugated secondary antibody, proteins were visualized with the SuperSignal West Pico Chemiluminescent Substrate (Pierce, Thermo Fisher Scientific Inc., Rockford, IL, USA) and autoradiography. Controls included the omission of the primary antibody, the use of an unrelated antibody, or assays using non-transfected cells.

### Computational Prediction Methods

InterProScan (http://www.ebi.ac.uk/Tools/pfa/iprscan/) and the HMMPred (http://toolkit.tuebingen.mpg.de/hhpred/) programs were used for protein signature. Signal peptide prediction was determined using the PSOTII (http://psort.hgc.jp/form2.html), Phobius (http://phobius.sbc.su.se/cgi-bin), and SecretomeP 2.0 (http://www.cbs.dtu.dk/services/SecretomeP/) programs. The program used for transmembrane domain prediction were TMPRED (http://www.ch.embnet.org/cgi-bin/TMPRED_form_parser), SOSUI (http://bp.nuap.nagoya-u.ac.jp/sosui/cgi-bin/adv_sosui.cgi), DAS (http://www.sbc.su.se/~miklos/DAS/tmdas.cgi) SPLIT (http://split.pmfst.hr/split/4/out/), TMHMM (http://www.cbs.dtu.dk/cgi-bin), MEMSAT, (http://bioinf.cs.ucl.ac.uk/psipred/), and Phobius (http://phobius.sbc.su.se/constrained.html).

### Immunofluorescence Assay

Trophozoites were washed with PBSm (1% growth medium in PBS, pH 7.4) and allowed to attach themselves to slides at 37°C. After fixation with 4% formaldehyde, the cells were washed and blocked with PBS containing 10% normal goat serum and 0.1% Triton X-100. The cells were then incubated with specific Abs in PBS containing 3% normal goat serum and 0.1% Triton-X100, followed by incubation with Alexa488-conjugated goat anti-mouse secondary antibody. For direct double staining, the anti-HA mAb (Sigma, St. Louis, MO) was labeled with Zenon Alexa Fluor 488 and was used to detect HA-tagged GlVps (final dilution of anti-HA 1∶500), while 9C9, 2F5 and anti-V5 mAbs were labeled with Zenon Alexa Fluor 555 (1∶200 final dilution), following the suggested protocol (Zenon Tricolor Mouse IgG1 Labeling Kit, Molecular Probes, Invitrogen Corporation, Carlsbad, CA). Controls included the omission of the primary antibody and the staining of wild-type cells. Finally, preparations were washed and mounted in Vectashield mounting medium. Fluorescence staining was visualized with a motorized FV1000 Olympus confocal microscope (Olympus UK Ltd, UK), using 63× or 100× oil immersion objectives (NA 1.32). The fluorochromes were excited using an argon laser at 488 nm and a krypton laser at 568 nm. DAPI was excited with ultraviolet light using a 364 nm Argon laser. Detector slits were configured to minimize any cross-talk between the channels. Differential interference contrast images were collected simultaneously with the fluorescence images, by the use of a transmitted light detector. Images were processed using FV10-ASW 1.4 Viewer and Adobe Photoshop 8.0 (Adobe Systems) software. The colocalization and deconvolution were performed using MetaMorph software (Molecular Devices, Silicon Valley, CA). Fluorescent images were observed with an inverted microscope (Carl Zeiss Axiovert 35M) equipped with epifluorescence and differential interference contrast (DIC) optics using a 100× oil immersion objective (Carl Zeiss) and were captured under regular fluorescence microscopy with a silicon-intensified target camera (SIT-C2400; Hamamatsu Phototonics, Bridgewater, NJ). The images were digitized directly into a Metamorph/Metafluor Image Processor (Universal Imaging Corporation, West Chester, PA).

### Quantitative colocalization analysis (QCA)

Confocal immunofluorescence microscopy and quantitative colocalization analysis were performed using Fiji image processing package (http://fiji.sc/wiki/index.php/Fiji). Background was corrected using the threshold value for all channels to remove background and noise levels completely. The Pearson's correlation coefficient (PC) and the overlap coefficient according to Manders (M) were examined. PC values range between −1.0 and 1.0, where 0 indicates no significant correlation and −1.0 indicates complete negative correlation. The M values are in the range from 0 to 1.0. If the image has overlap coefficient 0.5, it implies that 50% of both its objects, i.e. pixels, overlap. A value of zero means that there are no any overlapping pixels. This coefficient is not sensitive to the limitations of typical fluorescence imaging [27], [28], [29], [30]. According to the PC, the values indicating colocalization ranged from 0.5 to 1.0 while for the M colocalization is considered in the range from 0.6 to 1.0.

### Reverse transcription polymerase chain reaction (RT-PCR)

The total RNA from wild-type cells was isolated using Trizol reagent (Invitrogen), and a second purification was performed using the SV Total RNA Isolation System (Promega). RT-PCR was performed using One-step RT-PCR kit (Qiagen, Valencia, CA) as previously described [14]. For detection of endogenous *glvps* mRNA, the F1 and R1 primers were used to amplify the 1653 nt ORF, while the primers F2 (ATGAGCTGGCTCAAGAGTATGGTAGGACTGC) and R1 were used to obtain the 1490 nt fragment (from the 160 nt to the stop codon). The expression of the constitutive glutamate dehydrogenase enzyme (GDH) using previously described primers GDHf/GDHr [19] was performed for positive control. The DNA-contamination control was performed by adding the same primers at the PCR step of the RT-PCR reaction. These assays were performed three times in duplicate. For semi-quantitative RT-PCR, total RNA extracted from antisense transgenic trophozoites or trophozoites control containing empty vector, was diluted serially from 20 ng to 0.2 ng per reaction in a final reaction volume of 50 μl, and RT-PCR was carried out following the manufacturer's instructions. For the examination of µ1 downregulation, µ1 antisense and sense were amplified using the primers described in Touz et al. [13]. For *glvps* antisense amplification, the oligonucleotide ASf and ASr were added sequentially. For *glvps* sense amplification, F1 and R1 primers were added to OneStep the master mix. *acph* mRNA was determined using the primers described in [13]. Data normalization was carried out against the *gdh* endogenous reference gene transcript.

### Subcellular fractionation

Trophozoites were grown to logarithmic phase and washed twice in PBS, resuspended in cold hypotonic lysis buffer (10 mM TRIS-HCl, pH 7.5 plus protease inhibitors) and then incubated on ice for 5 min. The cell lysate was centrifuged in a refrigerate microfuge at 16 000x g at 4°C for 10 min. The pellet fraction was washed once with cold hypotonic lysis buffer, resuspended in sample buffer and incubated on ice for 25 min prior to taking for SDS-PAGE. Equivalent amounts of supernatant (SN), washing (W) and pellet (P) fractions were analyzed by immunoblotting [31].

### Digitonin or Triton X-100 Cell Permeabilization Followed by Digestion with Proteinase K (PK)

In tubes: GlVps-HA and wild-type trophozoites were collected and permeabilized, using either 0.1% of digitonin or 0.1% of Triton X-100 in PBS for 10 min on ice. After washing with PBS, the cells were treated with 5 µg/ml of proteinase K (PK) for 30 min on ice. Samples were inactivated by the addition of 1 mM phenylmethylsulfonyl fluoride (PMSF), treated with SDS-PAGE buffer, heated to 95°C, and cooled on ice. Samples were separated by SDS-PAGE before testing by immunoblotting. In slides: GlVps-HA and wild-type trophozoites were collected and attached to Poly-L-Lysine-covered slides at 37°C. After fixation with 4% formaldehyde, the cells were permeabilized by addition of 0.1% digitonin in PBS (Calbiochem). After two washes with PBS buffer, the trophozoites were treated for 5 min with varying concentrations of PK at 37°C. To terminate the PK reaction, 5 mM PMSF was added to all samples. The cells treated with PK were then incubated with specific Abs in PBS containing 3% normal goat serum and 0.1% Triton-X100, followed by incubation with Alexa488-conjugated goat anti-mouse secondary antibody. Controls included treatment with 0.1% of Triton X-100 or digitonin and Ab detection in PBS and PK treatment of non-permeabilized cells. After PBS washing, the samples were analyzed by IFA as described below.

### Yeast-two hybrid assay

The MATCHMAKER Two-Hybrid System was used following the manufacturer's recommended protocol (Clontech, Palo Alto, CA). The two-hybrid pGADT7-Rec(LEU2) vector (GAL4 transcription activation domain; AD) containing the sequences for *glvps* or Δ*glvps* (lacking the YQII motif sequence) were used as bait, while *acph* and *µ1* genes were inserted into the pGBKT7(TRP1) vector (GAL4 DNA binding domain; BD), yielding the **p**GlVps-AD, **p**GlVps_-YQII_ -AD, **p**AcPh-BD, and **p**µ1-BD vectors, respectively. The vectors **p**LRP-HA and **p**µ1-BD were used as control [32]. The AH109 transformants were cultured at 30°C for 4–5 days on plates with minimal medium lacking leucine and tryptophan (-L/-T) to test for positive transformation, or in the absence of leucine, tryptophan, and histidine (TDO, triple dropout medium) to study specific protein interactions as previously described [13]. High-stringency medium that also lacked adenine (QDO) was also used to test strong protein-protein interactions. Controls included the pESCP-AD/pµ1-BD interaction or pGlLRP-AD/pµ2-BD (protein-protein interaction control) [13], [32] and the **p**GlVps-AD/ **p**GBKT7 or **p**GADT7/ **p**GlVps-BD vector (autoactivation control).

## Results

### The subcellular localization of the soluble hydrolase AcPh

The localization of AcPh in the ER and PVs was in part determined by using diverse methodologies [11], [13]. In the present work, we aimed to acutely define the subcellular localization of AcPh together with its activity by a combination of complementary assays.

**Figure 1:**
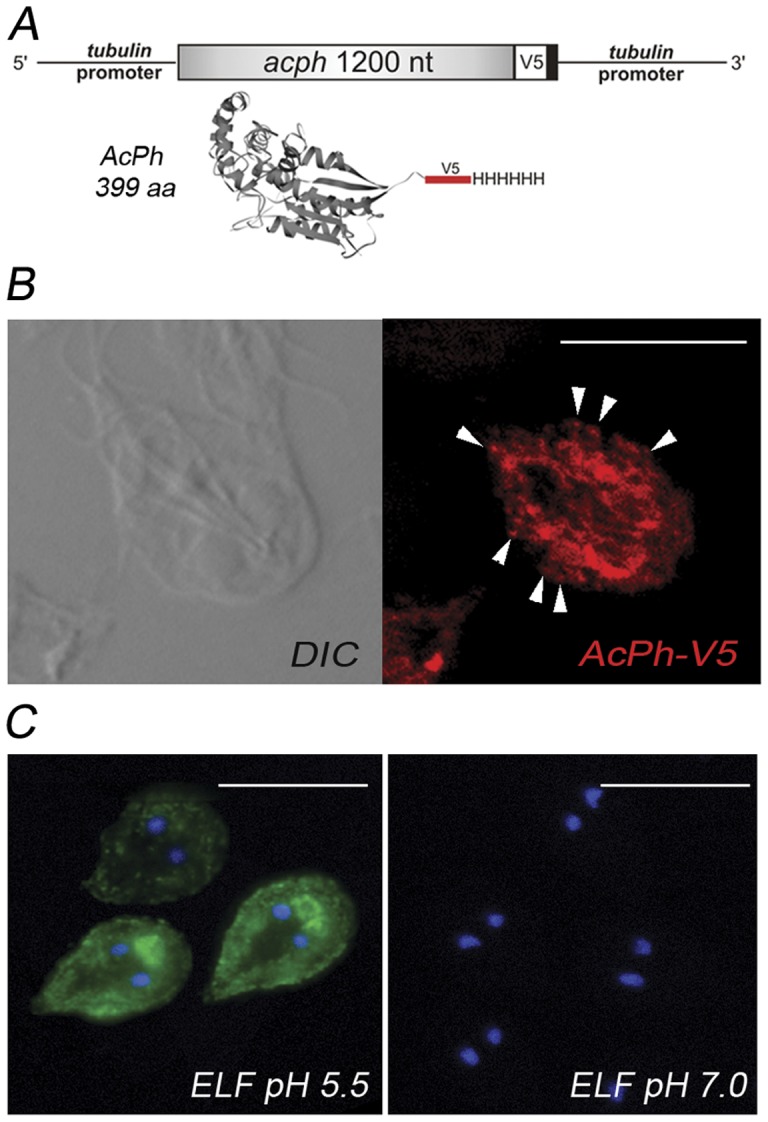
AcPh localization and activity. (A) Schematic representation of the *acph* gene containing the GGTAAGCCTATCCCTAACCCTCTCCTCGGTCTCGATTCTACGCGTA. CCGGT and CATCATCATCATCATCAT, coding to the V5 epitope and six histidine residues, respectively. A 3D reconstruction of the gene product tagged with V5-H6 using the hidden Markov models (HMMs) [84] and MODELLER [85] is also represented. (B) IFA and confocal microscopy show AcPh-V5 predominantly in the ER but also in the nuclear envelope and PVs (arrowheads). DIC: Differential interference contrast microscopy. (C) Acid phosphatase activity on the PVs and bare zone is observed by using the specific substrate ELF97 at pH 5.5. Alkaline phosphatase activity was not detected in trophozoites at pH ≥7.0. Nuclear DNA was labeled with 4′,6-diamidino-2-phenylindole (DAPI) (blue). Bar, 10 μm.

First, we constructed a C-terminus tagged AcPh containing a sequence that codified for a V5 epitope followed by six consecutive His (Figure 1A). Trophozoites transfected with the vector pTubAcPh-V5/H6pac were grown in culture medium, and subcellular distribution of AcPh-V5/H6 was analyzed by immunofluorescence. In agreement with previous reports on the enzyme cytochemical [11], [33] and HA-tagged AcPh localization [13], AcPh-V5/H6 was observed around the nuclei and close to the plasma membrane (Figure 1B). To assess where AcPh became active inside the cells, we used a phosphatase substrate ELF® 97, successfully used for the detection of acid and alkaline phosphatase activity by fluorescence microscopy [23], [24], [25], [26]. Fluorescence was observed upon substrate hydrolysis, producing a bright and photo-stable yellow-green fluorescent precipitate at the site of enzyme activity. This fluorescence accumulated in the PVs at pH 5.5 but was not detected at pH ≥7.0 (Figure 1C). Also, acid phosphatase activity was observed in the structure termed bare zone (BZ), located between both nuclei and suggested to be important in trophozoite attachment/detachment [34]. Recently, we found that it is composed by PV-like vacuoles (Touz et al., unpublished results). No difference in the fluorescence signal was observed between AcPh-V5/H6 transgenic and wild-type trophozoites in these assays (not shown).

Detailed AcPh-V5/H6 localization was obtained using immunoelectron microscopy of transgenic trophozoites (Figure S1). An illustrative electron micrograph of a *Giardia* trophozoite was shown to better describe the PVs and BZ (Figure S1A) [11], [35]. Labeling with anti-V5 mAb and the gold-labeled secondary antibody showed a distinctive signal, consistent with the localization of AcPh in these organelles (Figure S1B, C). No labeling was observed in treated wild-type trophozoites (Figure S1A, a). Altogether, these results suggested that, after synthesis in the ER, AcPh was delivered to the acidic compartment where it became active.

### Identification of the acid phosphatase receptor

**Figure 2:**
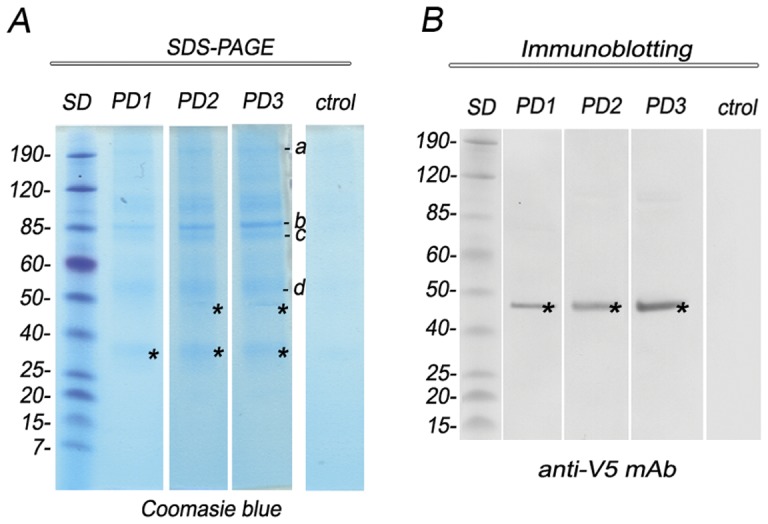
AcPh-V5H_6_ pulled-down associated proteins. (A) SDS-PAGE stained with Coomassie blue for total protein shows AcPh-V5H_6_-binding proteins from transgenic trophozoite extracts identified by affinity chromatography and mass spectrometry. PD1-3 are independent pull-down assays. The proteins identified are shown as: **a**: not determined; **b**: kinesin-like protein (GL50803_14070, GL50803_17264); **c**: Vacuolar protein sorting 35 (GL50803_23833); **d**: unknown protein (GL50803_28954). Asterisks (*) denote detection of AcPh. Control lane (Ctrol) shows no protein binding when wild-type trophozoites were used. (B) Immunoblotting shows the presence of AcPh-V5H_6_ in PD1-3 but not in control. Asterisks (*) denote detection of AcPh-V5H_6_ by using anti-V5 mAb. Molecular weights of protein standards (SD) in kDa are shown on the left.

The awareness that the soluble lysosomal protein AcPh might require a receptor-mediated sorting process, prompted us to initiate a search for a candidate using AcPh as bait. This result encouraged the use of AcPh-V5/H6 transgenic trophozoites to purify AcPh together with its associated proteins by pull-down assay. AcPh-V5/H6 was purified from transgenic cells using agarose-immobilized nickel ions that bound the string of histidine residues of AcPh. Linked proteins were eluted, analyzed by SDS-PAGE, and submitted to the Research Technologies Branch for Protein Identification (NIAID, NIH) for LC-MS/MS analysis. After three independent experiments, three proteins associated with AcPh were identified (Figure 2A, B and Table S1). Besides the identification of two protein bands corresponding to the AcPh, BLAST search and sequence analysis identified proteins involved in protein trafficking, including a sequence encoding to the kinesin-like protein (GL50803_14070), the Vacuolar protein sorting 35 (GL50803_23833), and a sequence corresponding to an hypothetical protein (GL50803_28954). Analysis of homologous sequences of GL50803_28954 using the different predictive programs did not provide information about the possible function of this protein. However, analysis of the protein sequences of 550 amino acids showed that this protein contained a lysosomal tyrosine-based motif YQII at its C-terminus, suggesting that it might be the receptor involved in binding and transport of AcPh to PVs in *Giardia*.

**Figure 3:**
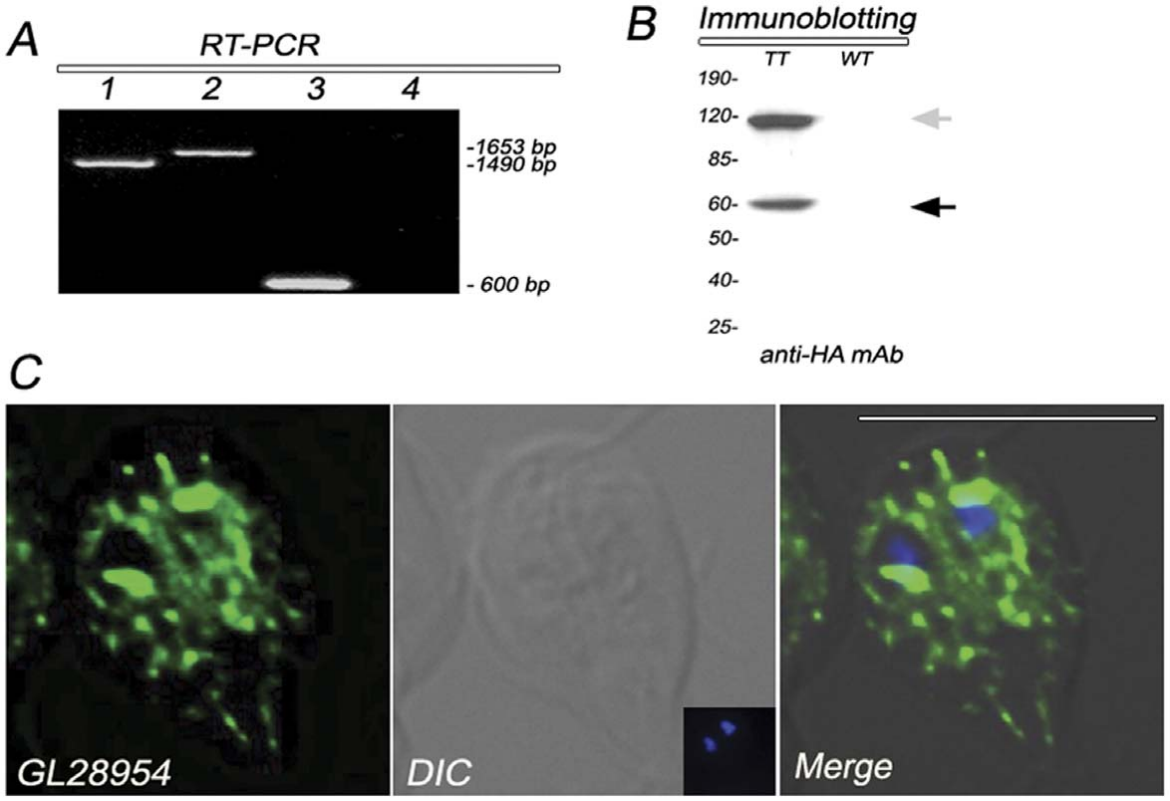
Expression of GL28954. (A) RT-PCR experiment show that the mRNA of GL28954 is expressed as predicted by the GiardiaDB. **1**: fragment of 1490 bp amplified using the primer pair F2/R1; **2**: the predicted 1653 bp ORF amplified using the primer pair F1/R1; **3**: expression of a *gdh* mRNA fragment was tested as positive control; **4**: DNA-contamination control. (B) Immunoblotting using anti-HA mAb shows the predicted band of 60 kDa for GL28954 (black arrow) but also a higher 120 kDa band that might correspond to GL28954 homodimer (gray arrow) in transgenic trophozoites (TT). Wild-type trophozoites (WT) do not show the presence of GL28954-HA. Relative molecular weights of protein standards (kDa) are indicated on the left. (C) IFA and confocal microscopy show the HA-tagged GL28954 mainly around the nuclei. DIC: Differential interference contrast microscopy. Nuclear DNA was labeled with DAPI (blue). Bar, 10 μm.

Analysis of its expression by RT-PCR showed that GL50803_28954 was expressed in growing trophozoites (Figure 3A). Because at that time the corresponding gene sequence was deprecated from the GDB, we decided to corroborate the start codon of the protein sequence by designing a set of primers that amplified a fragment of 1490 bp (from the ATG_160–162_) and another pair to amplify the whole predicted 1653 bp ORF (Figure 3A). These results clearly show that the putative ORF was expressed in the *Giardia* trophozoite assemblage A. Further, this assemblage A gene was undeprecated, based on synteny to assemblage B and E genomes. Immunoblotting revealed the expected band of around 60 kDa and a high band of around 120 kDa, suggesting that this fusion protein might form homodimers (Figure 3B) (see discussion). Immunofluorescence assays of constitutively expressed C-terminus HA-tagged GL50803_28954 showed this protein surrounding the nuclei and also close to the plasma membrane (Figure 3C).

### Topological analysis of GL50803_28954

Biostatistics analysis using Interproscan [36] showed sequence similarity between GL50803_28954 residues 240–482 and WD40 repeat-like superfamily SSF50978, which includes proteins possessing a fold of seven 4-stranded beta-sheet motifs (E-value of 2.7E-10). A secondary structure analysis using Jpred3 [37] concordantly predicts an abundance of residues with likelihood to form beta propellers in that residue stretch. HHPred also predicts similarity to WD40 repeat-like motif present in PDB entry 3emh with a probability of 91.4% and E-value of 6.4E-05. Sequence analysis using Phobius [38] indicates that this protein contains no N-terminal cleavable signal peptide to direct protein topology in the membrane. Regarding to the absence of a signal peptide in this protein, membrane proteins confined to sub-regions such as the smooth ER may be retained by forming oligomeric assemblies [39], [40]. Consequently, such proteins may not need structural retention motifs [41]. We expect that GlVps follows a mechanism where membrane proteins confined to sub-regions such as the smooth ER may be retained by forming oligomeric assemblies to enter to the secretory pathway. We also tested whether GlVps can be exported without a classical N-terminal signal peptide using the sequence based method SecretomeP 2.0, for prediction of mammalian and bacterial secretory proteins targeted to the non-classical secretory pathway. This method was also capable of identify GlVps as a protein that follows a signal peptide independent secretion pathway with a NN-score of 0.791 (non-classically secreted proteins should obtain an NN-score exceeding the normal threshold of 0.5) [42], [43].

**Figure 4:**
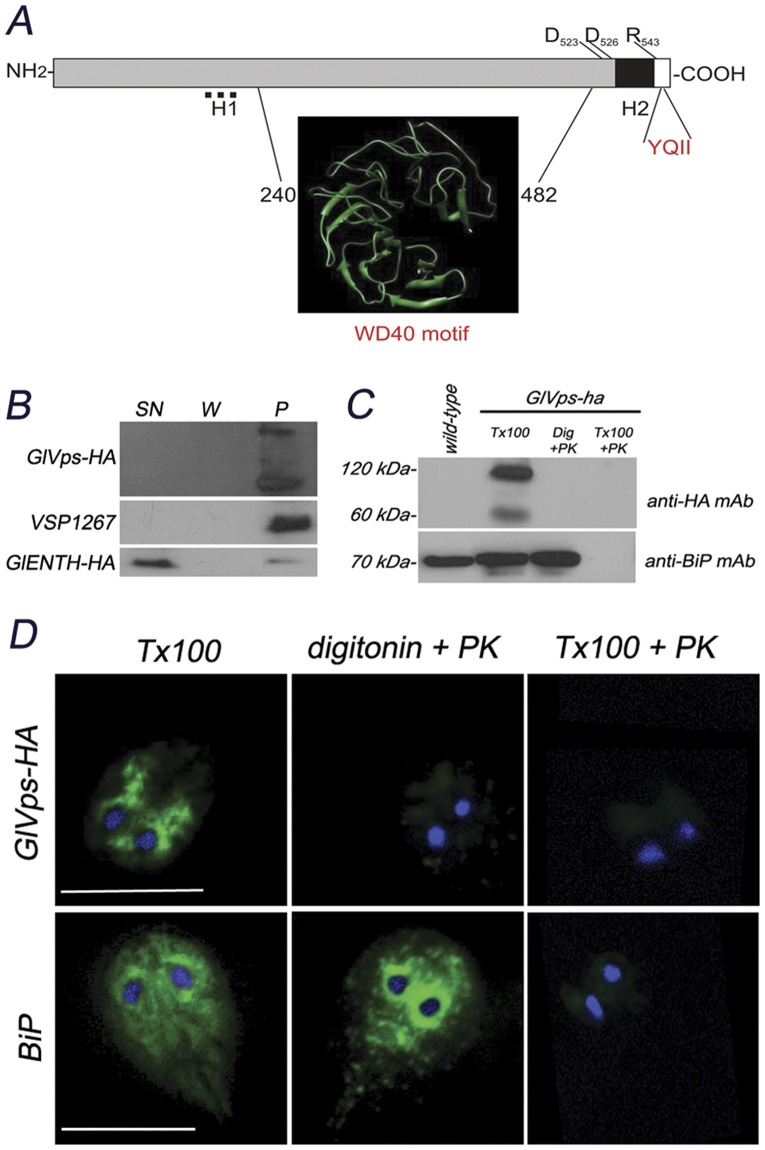
The *Giardia lamblia* Vacuolar protein sorting (GlVps) topology analysis suggests an N_lumenal_/C_cytoplasmic_ localization. (A) Schematic representation of GlVps. The presence of a transmembrane domain (in black) flanked by the TM-stop residues (R_543_, D_523_, and D_526_) is shown. The unlikely hydrophobic motif H1 (residues 143 to 167) is also depicted in dotted bar. One WD40 protein-binding domain (3D structure) between the residues 240–482 at the N-terminus (in gray) and the cytoplasmic tail (in white) containing the YQII lysosomal motif are shown in the diagram. (B) *GlVps-ha* transgenic cells were grown and lysed under hypotonic conditions. Supernatant (SN) and pellet (P) fractions were recovered, concentrated to normalize loading and analyzed by immunoblotting. GlVps-HA and VSP1267 are restricted to the membrane-bound population in the pellet (upper and middle panel). The cytosolic GlENTH-HA protein (Feliziani and Touz, unpublished results) is mainly present in the cytosolic fraction. W: soluble proteins after washing. Anti-HA mAb was used for GlVps-HA and GlENTH-HA and 5C1 mAb for VSP1267. (C) Immunoblotting after proteinase K (PK) assay shows the protection of BiP but not GlVps-HA after membrane permeabilization by digitonin (Dig+PK). Permeabilization with Triton X-100 previous to PK treatment shows both BiP and GlVps-HA degradation (Tx100+PK). Permeabilization with Triton X-100 without PK addition shows the presence of both proteins (Tx100). In these assays, wild-type trophozoites not expressing GLVps-HA and GlVps-HA transgenic cells were used. Relative molecular weights of protein standards (kDa) are indicated on the left. (D) IFA and epifluorescence microscopy after 50 µg PK assay confirms that the C-terminal portion of GlVps is unprotected from protease, revealing the cytoplasmic orientation of its C-terminus (digitonin+PK, top panel). Conversely, the lumenal ER-protein BiP was not processed by the PK (digitonin+PK, bottom panel) and was detected in the ER. Degradation of BiP and GlVps-HA was observed in the control of cells permeabilized with Triton X-100 previous PK treatment (Tx100+PK) but not in the control, where the cells were treated with Triton X-100 or digitonin without PK addition (Tx100 – digitonin). Anti-BiP or anti-HA mAb were used in the assay shown in (B) and (C) to detect BiP or GlVps, respectively. GlVps-HA transgenic cells were used in this experiment. Individual images were processed in the same way. Nuclear DNA was labeled with 4′,6-diamidino-2-phenylindole (DAPI) (blue). Bar, 10 μm.

Besides Phobius, numerous topology-predicting algorithms identified two hydrophobic motifs (H1, residues 143 to 167; H2, residues 526 to 542) as potential TM domains, suggesting that GL50803_28954 utilizes these motifs as signal-anchor sequences to direct a polytopic membrane topology. However, these algorithms disregard the presence of charged D and R residues inside H1, making this region an uncertain membrane domain. On the other hand, it was extensively shown that positively charged residues are commonly observed at the end regions of transmembrane helices, particularly on the cytoplasmic side [44], [45], [46], as is the case for the R_543_ flanking H2. Moreover, H2 is preceded by two conserved aspartate residues (D_523_, D_526_), probably acting as TM-stop for luminal phase, indicating the importance of amphiphilic residues for stabilization of transmembrane helices at the membrane-water interphase [47]. Since the WD40 repeat motif should face the luminal site and the H2 is consistent with the “positive-inside rule” of membrane protein topology, we predicted an N_lumenal_/C_cytoplasmic_ orientation of this protein, with the YQII exposed to the cytosol (Figure 4A). Running Phobius with the restriction that the portion containing the WD-40 motif should be luminal, the probability for GlVps N_lumenal_/C_cytoplasmic_ orientation is from 0.8 to 1 with a probability >0.75 being significant [48]. This predicted topology would be similar to PEP1 (vps10p) topology, which displays a large luminal domain containing several BNR (bacterial neuraminidase repeat) that form a beta propeller domain, a transmembrane domain, and a short cytoplasmic tail displaying an adaptin interaction motif [5], [49], [50]. Thus, we named this protein *Giardia lamblia* Vacuolar protein sorting (GlVps).

An initial subcellular fractionation using hypotonic lysis of GlVps-HA transgenic trophozoites indicated that GlVps was present as a membrane-associated population similar to the type-I membrane protein VSP1267 (Figure 4B). Further studies using epitope-tagging of GlVps at the C-terminus and proteinase K (PK) protection assay confirmed a bitopic N_lumenal_/C_cytoplasmic_ topology. This assay uses the restricted proteolytic digestibility of proteins to reveal the intramembrane orientation of proteins residing in organelles as diverse as the Golgi apparatus, the ER, peroxisomes, mitochondria, among others [51]. As shown in Figure 4C, the intact, the luminal (ER)-resident chaperone BiP/GRP78 was protected by ER-microsomal membranes when treated with digitonin (which selectively permeabilized the plasma membrane) and proteinase K (Figure 4C, lane 3, lower panel), while the HA epitope of GlVps-HA was not (Figure 4C, line 3, upper panel). GlVps-HA transgenic and wild-type cells, permeabilized by 1% Triton X-100 without addition of PK, were used for proteolysis control (Figure 4D, Tx100). No obvious reduction in size of the GLVps-HA protein was observed. Identical results were obtained by analysis of fluorescent signals of cells permeabilized by digitonin and subsequently treated with proteinase K (Figure 4D, digitonin+PK). As expected, in both assays no signal was detected when proteinase K was used after Triton X-100 permeabilization (Figure 4C, D, Tx100+PK). Control of GlVps-HA transgenic cells permeabilized by 1% Triton X-100 or digitonin without addition of PK examined by indirect immunofluorescence, showed the presence of both proteins (Figure 4D, Tx100 – digitonin). Addition of PK alone did not present proteolytic digestibility (not shown). It was thus possible to determine that indeed the C-terminus of GlVps-HA faces the cytosol.

### Overexpressed GlVps-HA accumulates in the ER and to some extent in the PVs

**Figure 5:**
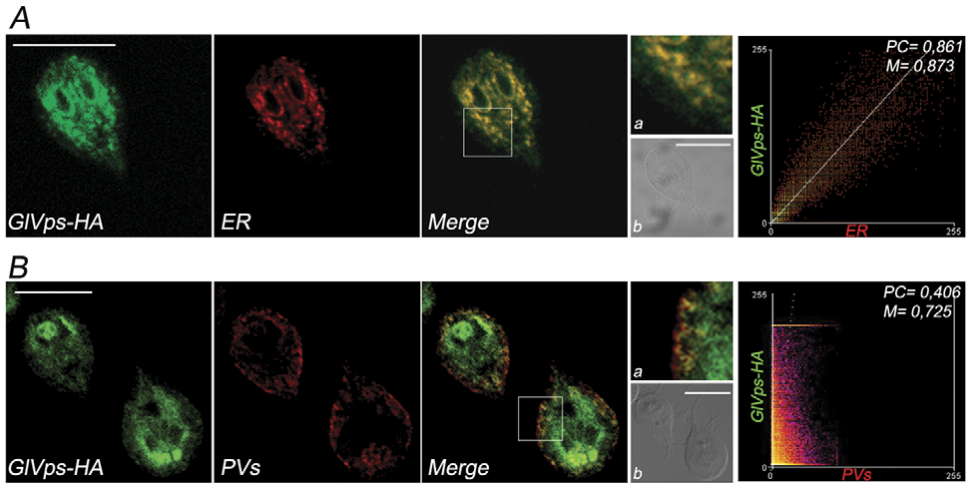
Subcellular distribution of GlVps. (A) Direct IFA and confocal microscopy show that GlVps-HA (green) partially colocalizes with the ER-resident chaperone BiP (red) in the ER (Merge in yellow). Inset magnifies a region of the cell and shows the green and red fluorescence in the ER (a). Differential interference contrast microscopy (b) is shown as insert. Scatter plot of the two labels confirms the colocalization (right panel). (B) Direct IFA and confocal microscopy using the 2F5 mAb that detect the μ2 subunit of AP2 in the PVs (red) and anti-HA mAb to detect GlVps-HA (green), show the presence of GlVps-HA in some PVs. Inset magnifies a region of the cell where the green and red fluorescence partially overlap (a). Differential interference contrast microscopy (b) is shown as insert. Bar, 10 μm. Scatter plot (panel on the right) correspond to the colocalization analysis. Pearson's coefficient (PC). Manders' Overlap coefficient (M).

Trophozoites expressing GlVps-HA exhibited a noticeable fluorescence around the nuclei, suggesting that this receptor might be retained in the ER. An antibody against the (ER)-marker BiP [15], was employed to visualize this compartment and to perform colocalization analysis showing that BiP colocalized with GlVps-HA in fixed cells (Figure 5A, top row). Detailed observations revealed that GlVps-HA localized in particular zones of the ER, in agreement with observations that the protein sorting station in *Giardia* might take place in distinct ER domains [52], [53]. Quantitative colocalization analysis demonstrated high degree of colocalization between BiP and GlVps-HA (Figure 5A, Scatter plot on the right). Scatter plots estimate the amount of detected fluorescence based on localization of GlVps-HA (green, y-axis) and BiP (red, x-axis). Colocalized pixels (yellow) are located along the diagonal of the scatter gram. The scatter plots indicate a yellow monopartite diagonal scatter pattern, which verifies the colocalization of both proteins in the ER. This observation was supported by the results of coefficients calculations: the Pearson's correlation coefficient (PC) and the overlap coefficient according to Manders (M) were 0.861 and 0.863, respectively. Thus, the results of coefficients calculations helped to find out more about the localization of GlVps-HA than it was possible to do using morphological experiments only.

The receptor-dependent delivery of AcPh to the PVs proposed that GlVps might cycle between these sorting points to the vacuoles. Direct immunofluorescence revealed that there was only a partial colocalization of GlVps-HA with the PV marker adaptor protein 2 (AP2) in the PVs (Figure 5B). PC decreased to 0.406 with M of 0.725 showing that this partial colocalization was real (Figure 5B, Scatter plot on the right).

### The YQII motif of GlVps is required for receptor localization and interaction with AP1

**Figure 6:**
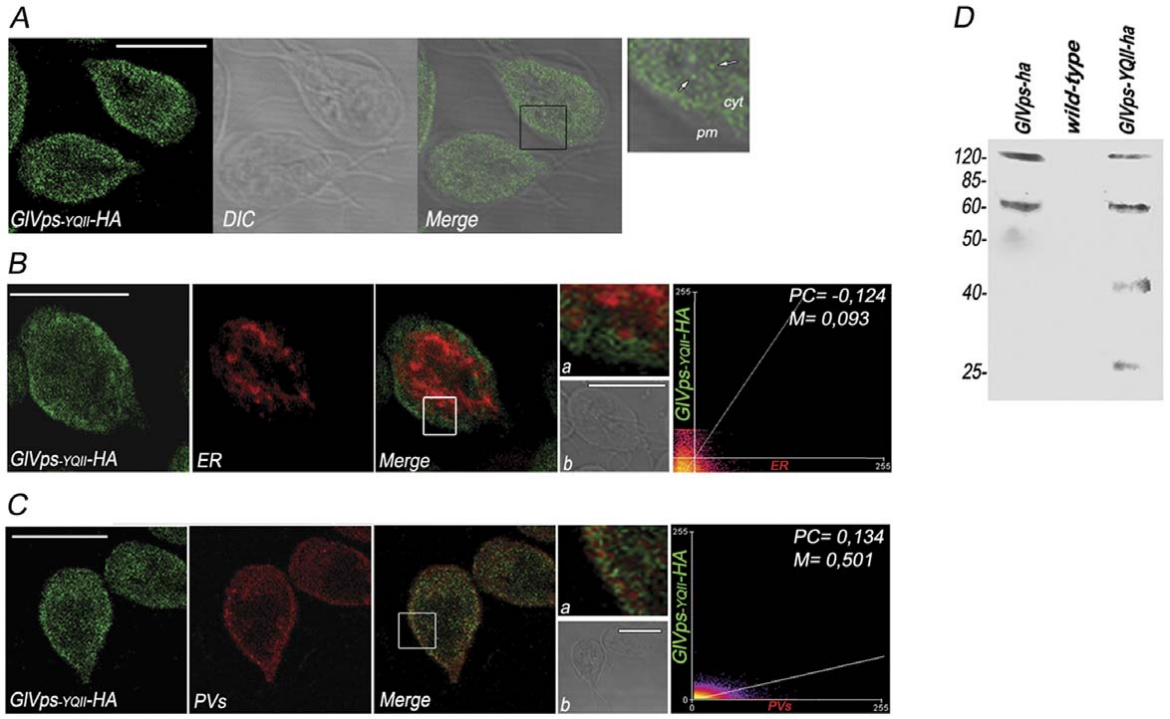
The YQII motif of GlVps contributes to receptor stabilization. (A) GlVps_-YQII_–HA (green) is observed in the cytosol (cyt) of trophozoites probable in small vesicles (white arrows in insert) by IFA and confocal microscopy. pm: plasma membrane. (B) GlVps_-YQII_–HA (green) do not colocalizes with BiP (red) in the ER (Merge). Inset (a) magnifies a region of the cell and shows that the green and red fluorescence are well separated. Differential interference contrast microscopy (b) is shown as insert. Scatter plot (panel on the left) correspond to the colocalization analysis. (C) Partial colocalization of GlVps_-YQII_–HA (green) and μ2 (red) is observed in the PV region (Merge). Inset (a) magnifies a region of the cell where the green and red fluorescence partially overlap in the PVs. Differential interference contrast microscopy (b) is shown as insert. Bars, 10 μm. Scatter plot of the two labels shows the colocalization (left panel). Pearson's coefficient (PC). Manders' Overlap coefficient (M). (D) GlVps-HA and GlVps_-YQII_–HA are detected by immunoblotting using anti-HA mAb in *GlVps-ha*, *GlVps_-YQII_–ha* trophozoites, respectively. No detection of these receptors was observed in wild-type cells. Proteolytic processing is observed for GlVps_-YQII_–HA in comparison with GlVps–HA. Relative molecular weights of protein standards (kDa) are indicated on the left.

The intracellular trafficking of the yeast Vps10p is mediated by its cytosolic domain [54]. This domain seems to interact with components of the lysosomal delivery system (i.e., clathrin-associated adaptor molecules Gga1p and Gga2p) to direct loaded receptors into transport vesicles destined for the endosome [55]. Similar in structure to yeast Vpsl0p, GlVps is an integral membrane protein but with a short RTVYQIIV carboxy-terminal amino acids exposed to the cytoplasm. To examine the functional requirement of GlVps' cytoplasmic domain, a mutant was constructed in which a stop codon was inserted into the sequence of GlVps after nucleotide 1634. This resulted in the production of a truncated GlVps protein lacking the YQII lysosomal motif but leaving the transmembrane domain intact, including a few charged amino acids on the cytoplasmic side of the membrane to properly anchor the protein. This new GlVps_-YQII_-HA construct was introduced in wild-type trophozoites yielding the *GlVps_-YQII_* transgenic cells. A significant amount of this mutant was detected at the cytoplasm in a punctate pattern (Figure 6A). Conversely to GlVps-HA, GlVps_-YQII_-HA was not retained in the ER as shown by the double immunostaining using anti-BiP and anti-HA mAbs (Figure 6B). The coefficients P = −0.124 and M = 0.093 indicated no colocalization between BiP and GlVps_-YQII_-HA in fixed trophozoites (Figure 6B, Scatter plot on the right). Labeling of GlVps_-YQII_-HA is observed in the PVs (Figure 6C). Colocalization analysis between GlVps_-YQII_-HA and AP2 showed a moderated degree of colocalization with P = 0,134 and M = 0,501. These observations indicate that the YQII residues affected GlVps trafficking. When *GlVps10*, *wild-type*, and *GlVps_-YQII_* trophozoites were analyzed by immunoblotting, degradation of the mutant receptor GlVps_-YQII_-HA was observed (Figure 6D). To analyze whether the expression of the transgenic cells affected *Giardia* growth, time-course curves were performed using *GlVps10*, *wild-type*, and *GlVps_-YQII_* cells and no significant effect or cell deterioration was observed at 48 h of culturing (not shown).

**Figure 7:**
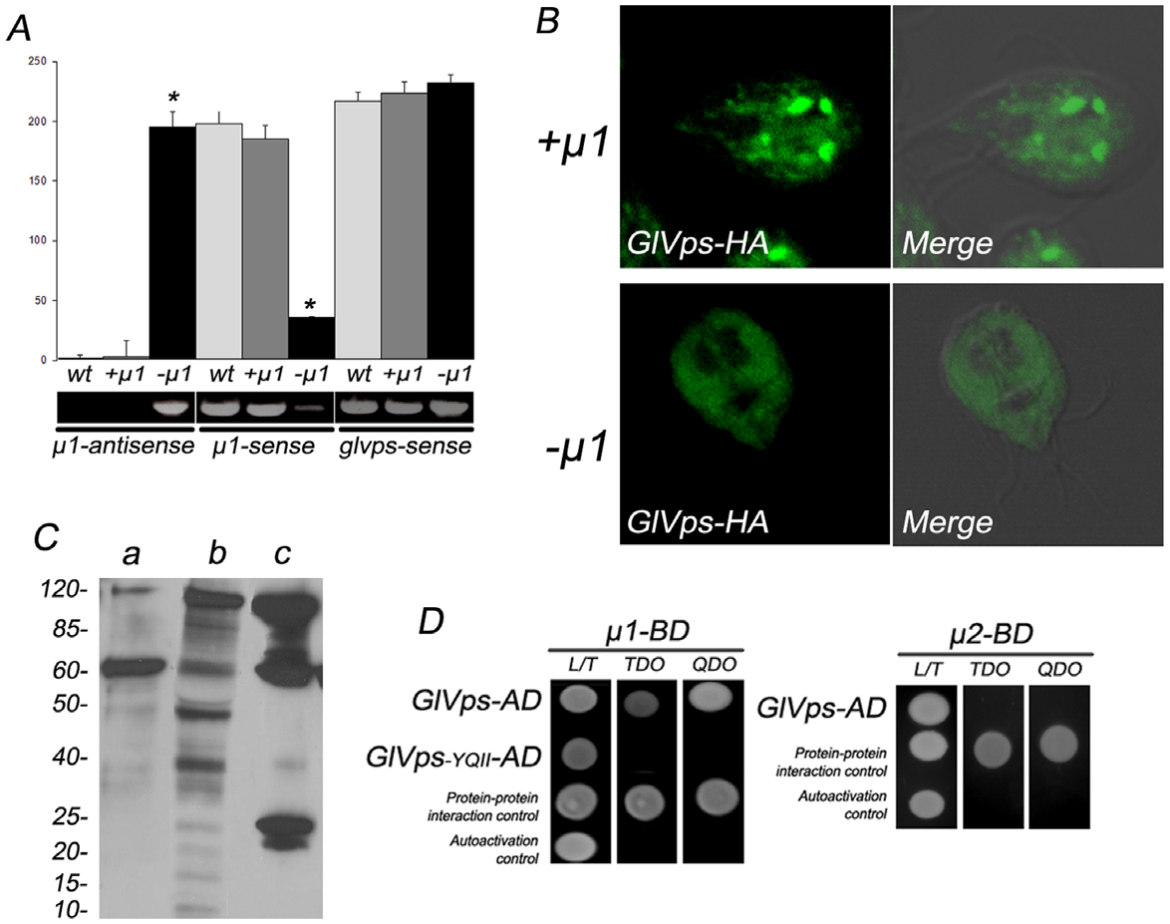
GlVps and the medium subunit of AP1 interact via the YQII motif. (A) Densitometric assessment of one representative RT–PCR experiment shown on bottom. The amount of 1000 nt antisense RNA from the vector is only observed in −µ1 trophozoites. Reduction of endogenous μ1 mRNA levels is observed in −µ1, but not in +µ1 or wild-type cells (wt). Similar expression of *glvps* mRNA in wild-type, +µ1 and −µ1 cells was observed. (*p<0,0001). (B) GlVps-HA is observed in the cytoplasm in µ1-depleted cells. In cell expressing µ1 (+µ1), GlVps-HA possesses a reticular-perinuclear distribution. Merge panels of green fluorescence and differential interference contrast microscopy for +µ1 and −µ1 trophozoites are shown. Bar, 10 μm. (C) GlVps-HA is detected by immunoblotting using anti-HA mAb in +µ1 (a) and −µ1 (b) trophozoites. The proteolytic processing of GlVps-HA observed in −µ1 trophozoites, differs from the processing of GlVPS_-YQII_-HA in cells expressing µ1 (c). Relative molecular weights of protein standards (kDa) are indicated on the left. (D) The yeast two-hybrid assay demonstrates that GlVps (GlVps-AD) but not GlVps_-YQII_ (GlVps-AD lacking the lysosomal motif) interacts with μ1 (μ1-BD) (left panel). GlVps (GlVps-AD) does not interact with the μ2 subunit of AP2 (μ2-BD) (right panel). Interaction is noticed by the growth of yeast colonies in plates lacking tryptophan, leucine and histidine [TDO (triple-dropout medium) plates] and in the high-stringency medium that also lacked adenine (QDO). Controls of the methodology include testing of pESCP-AD/pµ1-BD or pGlLRP-AD/pµ2-BD (protein-protein interaction) and pGlVps-AD/pGBKT7 (autoactivation).

We recently showed that transport along the vacuolar pathway requires clathrin and the adaptors AP1 or AP2, with AP1 being involved in the forward lysosomal protein trafficking to the PVs, while AP2 participates in endocytosis [56]. Unlike yeast and mammalian cells, *Giardia* does not contain GGAs homologous proteins, making AP1 the primary candidate for GlVps transport. To analyze whether AP1 is involved in GlVps trafficking, *dsRNA-µa* transgenic trophozoites [13] were cotransfected with the plasmid expressing GlVps-HA. Thus, we were able to analyze the expression and localization of GlVps-HA in trophozoites containing the µ1 subunit of AP1 (+μ1) and in trophozoites expressing a reduced amount of μ1 (−μ1). Densitometric analysis of RT–PCR experiments showed that, when these trophozoites were induced with 10 μg/ml of tetracycline, the μ1-antisense RNA was present in −μ1 but not in +μ1 or wild-type (wt) cells (Figure 7A). Furthermore, a significant reduction of the endogenous μ1 was observed in −μ1 trophozoites compared with +μ1 or wt cells when the 3′ endogenous segment of μ1 was tested [13], supporting our previous results showing that production of μ1-dsRNA successfully diminished the transcript levels of μ1 mRNA (Figure 7A). No alteration on the *glvps* mRNA expression was observed between the −µ1, +µ1 or wt cells (Figure 7A). However, IFA and confocal microscopy showed that GlVps-HA was present as a distinctive punctuate pattern in −µ1 cells whereas the perinuclear/reticular localization of GlVps-HA in +µ1 cells was conserved (Figure 7B). Immunoblotting showed that degradation of the receptor GlVps-HA occurred in the transgenic −µ1 cells (Figure 7C, line b) when compared with +µ1 cells (Figure 7C, line a) and differed from the processing observed for GlVps_-YQII_-HA in cells containing µ1 (Figure 7C, line c and Figure 6D). Evidence of an interaction between the GlVps and AP1 was obtained in the yeast two-hybrid system (YTH). Initial analysis demonstrated that GlVps strongly interacted with µ1 (Figure 7D, top panels). Moreover, GlVps_-YQII_ (lacking the lysosomal YQII sorting motif) provided no response (Figure 7D, left panels). When the interaction between GlVps and µ2 was tested, no positive results were obtained (Figure 7D, right panels). Altogether these results suggest that GlVps protein trafficking depends on its tyrosine-motif, which is capable of binding AP1 but not AP2 for this purpose.

### GlVps is the presumed AcPh receptor

**Figure 8:**
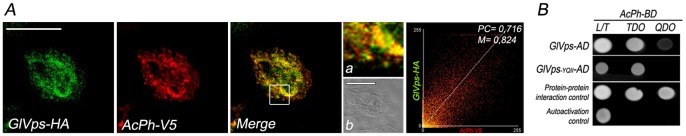
GlVps and AcPh colocalized throughout the lysosomal pathway. (A) Direct IFA and confocal microscopy show the colocalization (Merge) of GlVps (green) and AcPh-V5H_6_ (red) using directed labeled anti-HA and anti-V5, respectively. Inset magnifies a region of the cell and shows colocalization of the green and red fluorescence in yellow (a). Differential interference contrast microscopy (b) is shown as insert. Scatter plot of the two labels confirms the colocalization (right panel). Bar, 10 μm. (B) AcPh/GlVps and AcPh/GlVps_-YQII_ interaction was detected by the ability of yeast cells (AH109) to grow on selective plates TDO. No interaction was observed in the high-stringency QDO medium. Controls of the methodology include testing of pESCP-AD/pµ1-BD (protein-protein interaction) and pGlVps-AD/pGBKT7 (autoactivation).

**Figure 9:**
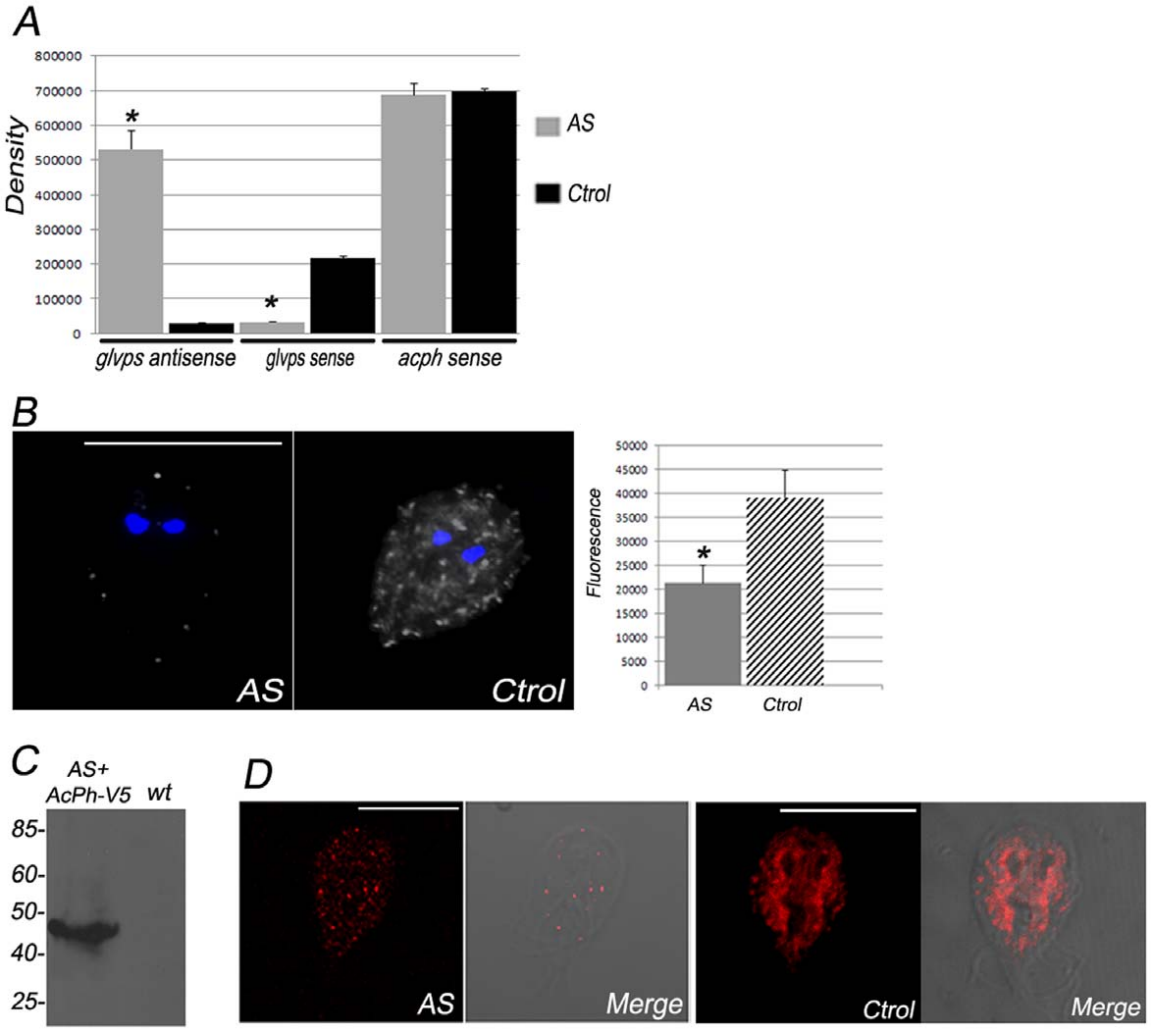
GlVps depletion affects AcPh localization and activity. (A) Bars indicate densitometric assessment of one representative RT-PCR experiment using 10 ng of total RNA from *GlVps-antisense* (AS) and *empty* (Ctrol) transgenic trophozoites. The densitometric analysis shows the production of *glvps*-antisense RNA in AS trophozoites but not in Ctrol cells. On the contrary, the *glvps* transcripts are highly reduced only in AS cells. *acph* mRNA was not altered in these cells. All transcripts were normalized against *gdh* endogenous control before graphic construction. Results are the means ± S.D. of three independent experiments (*p<0,0001). (B) By using the specific substrate ELF97 at pH 5.5, a notably reduction of acid phosphatase activity is observed in *GlVps-antisense* (AS) compared with *empty* (Ctrol) transgenic trophozoites. Nuclear DNA was labeled with DAPI (blue). One representative picture is shown. Bar, 10 μm. The graph on the left shows the quantitative fluorescent measurements of ELF97 (acid phosphatase activity). A significant decrease in mean fluorescence in AS cells is observed when compared with Ctrol cells (*p<0,0001). The mean fluorescence of all the acidic vesicles was calculated within each cell. Results are the means ± S.D. of 100 independent cells/group. (C) Crude protein extract from equal numbers of *GlVps-antisense+AcPh-V5* transgenic and *wild-type* (wt) trophozoites was separated by SDS–PAGE and analyzed by immunoblotting using anti-V5 mAb. Relative molecular weights of protein standards (kDa) are indicated on the left. (D) AcPh-V5 is mislocalized in *GlVps-antisense+AcPh-V5* (AS) transgenic trophozoites compared with control *AcPh-V5* transgenic trophozoites (Ctrol).

When GlVps-HA was co-expressed with AcPh-V5 and analyzed with IFA and confocal microscopy, we observed that GlVps-HA and AcPh-V5 colocalized around the nuclei and also in some PVs (Figure 8A). Correlation values (P = 0,716 and M = 0,824) indicate a significant degree of colocalization for both proteins. To test directly for the association of GlVps and AcPh, we performed yeast two-hybrid assays. After co-transformation and colony growth assays, we observed that GlVps and GlVps_-YQII_ certainly interacted with AcPh, allowing the yeast reporter to grow in stringent growth medium (Fig 8B). However, this interaction seemed not to be strong, since no colonies were obtained in high-stringency growth medium. These results, in addition to the pull-down findings, suggest that GlVps and AcPh interact and may be transported together toward the PVs. The role of GlVps in transport was further tested by inhibition of GlVps expression by antisense production. Semiquantitative reverse transcription-PCR revealed an increase of the GlVps-antisense RNA production as well as a fivefold decrease in GlVps-sense RNA analysis compared with control containing empty vector (Figure 9A). No variation on the native *acph* mRNA was observed when GlVps was depleted (Figure 9A). To test the effect of GlVps down-regulation in the localization and activity of AcPh the phosphatase substrate ELF®97 was used. We observed that the activity of AcPh was dramatically reduced in *GlVps-antisense* transgenic trophozoites compared with control containing empty vector (Figure 9B). These data suggested that GlVps participates in the transport of AcPh to the PVs where the enzyme became active. To test the effect of GlVps down-regulation in the localization of AcPh, cotransfection of *GlVps-antisense* transgenic trophozoites with the plasmids expressing AcPh-V5 was performed and the correct expression of AcPh-V5 observed by immunoblotting (Figure 9C). By IFA and confocal microscopy, we showed that there was a different pattern of AcPh-V5 localization in *GlVps-antisense+AcPh-V5* transgenic trophozoites compared with its localization in *AcPh-V5* transgenic trophozoites (Figure 9D).

## Discussion

Enzyme cytochemical localization for AcPh activity in lysosomal PVs as well as in the endoplasmic reticulum was first described in 1987 [33]. Although AcPh function during trophozoite growth and encystation remains in question, it was shown that dephosphorylation of the cyst wall proteins by AcPh is a required step for *Giardia* excystation [57]. Nevertheless, it is now clear that acid phosphatase activity and localization depend on a particular lysosomal protein trafficking, with exclusion of AcPh from other secretory pathways such as the constitutive surface membrane or regulated secretory proteins [11], [13], [58]. In the current report, we identified a membrane protein (GlVps) that fulfills the basic requirements for an AcPh receptor in *Giardia*. This contains a WD40-domain that was shown to participate in protein-protein interaction, and a C-terminal cytosolic tail containing a sequence signal for binding to sorting adaptor proteins. GlVps is present in the ER and rarely in PVs and colocalizes with AcPh. Depletion of GlVps or µ1 [13] modified the activity and the location of AcPh, with µ1 knock-down producing a missorting of GlVps to the cytoplasm.

In other parasites, acid phosphatase has been proposed as a virulence factor [59], [60], [61], [62]. However, the AcPh from *Giardia* does not seem to be secreted, even in contact with intestinal cells [63]. It is possible that its function may be restricted in order to accomplish the excystation process, but it is striking that this hydrolase was invariably expressed during the entire life cycle. Unlike acid phosphatases from other microorganisms, AcPh is a soluble protein that needs to be specifically sorted to the PVs by a receptor. Therefore, the finding that GlVps and AcPh interact and also colocalize in the ER and in the PVs area suggests that they might travel together from the recruitment and sorting site (around the nuclei) to the lysosomal PVs. Like AcPh, the lysosomal cathepsin B-like cysteine proteases GlCP1, GlCP2, and GlCP3 [64], [65] were observed in both the peripheral TVN (tubulovesicular network) and the perinuclear region. However, cathepsin activity, identified by in situ cleavage of the MNA derivatized peptide substrate, was localized to the same region of the cell and [65] excluded from the PVs and the BZ as was shown in this report for AcPh activity. Thus, GlVps might unlikely be the receptor for all the soluble hydrolases present in *Giardia* trophozoites.

Following the mechanisms described for yeast and mammalian cells, GlVps might dissociate from AcPh when they reach the acidic pH of the PVs and the receptor recycles back via the retromer complex (reviewed by [66]). In fact, the cargo-selection subunit GlVps35, homologous to Vps35 of the yeast retromer, was pulled-down together with AcPh and GlVps [67]. Since there is no distinguished prevacuolar compartment in *Giardia,* it is possible that this process of docking-dissociation-recycling occurs almost at the same time, with the AcPh-GlVps-GlVps35 interaction being possible. Experiments concentrating on the functional participation of GlVps35 and the *Giardia* retromer complex in GlVps recycling are currently underway to test this hypothesis. Also, a combination of genetic and biochemical approaches analyzing the interaction between AcPh and kinesin might contribute to the elucidation of the link between lysosomal protein trafficking and microtubule tracks, where the kinesin-like motor protein might be involved [68].

The identification of the WD40 domain in GlVps together with its subcellular localization suggest that it might behave like the human WD40 protein, WIPI49 (later termed WIPI1) or the yeast-orthologous Vps18p, with the capacity to regulate endosomal trafficking of proteins and autophagosome formation [69], [70]. Besides the presence of the immunoreactive band at the size of GlVps-HA, a higher band, probably corresponding to the size of GlVps homodimer, was observed, suggesting that this protein might interact with itself, as has been shown for several proteins containing one or more WD40 domains [71], [72]. However, we cannot exclude the possibility that the higher molecular weight complex also contains a different protein of similar size that interacts with GlVps. The absence of a cleavable N-terminal signal peptide, and the use of a signal-anchor sequence that directs translocation of the N-terminal domain across the membrane, designate GlVps as a type III-like membrane protein [46]. In the case of GlVps, the orientation of signal-anchor proteins in the ER membrane seemed to be dictated to a large extent by the charge distribution in the residues that flank either side of the TM domain, with a net internal positive charge favoring an N_lumenal_/C_cytoplasmic_ topology.

Transport of the yeast Vps10p along the prevacuolar endosome-like/vacuolar pathway requires clathrin and the adaptors Gga1p and Gga2p [73]. Deletion of both genes impairs proteolytic processing of the inactive precursors of the vacuolar hydrolase CPY [74]. Interestingly, Vps10p does not have the canonical DXXLL signals that are involved in the recognition by the Ggas, with the possibility that the yeast Ggas might recognize a different sorting motif [55]. Moreover, while Vps10p contains two aromatic-based signals, YSSL and FYVF, in its cytoplasmic tail, the mutation of individual AP1 subunits or deletion of the whole complex in yeast results in no measurable protein-trafficking phenotype [75]. We showed that the cytoplasmic YQII motif of GlVps is essential for the proper localization and stability of this receptor. Because this lysosomal motif was critical for GlVps-μ1 interaction, we presumed that the AP1 complex together with clathrin might be participating in the trafficking of the receptor, similar to the transport of the lysosomal membrane protein ESCP [13]. YQII-deleted GlVps and GlVps from µ1 depleted trophozoites were proteolytically processed, suggesting that either the absence of the sorting motif or the lack of the adaptor counterpart this receptor might be degraded in the PVs or in the cytoplasm by proteasome. It is now known that integral membrane proteins with misfolded cytoplasmic domains go through ubiquitin and proteasome-mediated degradation [76], [77], [78]. Further investigations are needed to clarify this observation.

Again and again, analysis of *Giardia* protein trafficking showed many particularities, although a minimal machinery is still conserved. Similar to what happens in yeast, AcPh-GlVps interaction seems to be independent of oligosaccharides since protein glycosylation is controversial in this parasite, as there is no definitive evidence for either *N*- or O-glycosylation in any *Giardia* protein [79], [80]. Analysis of lysosomal proteins like AcPh and GlVps disclose some interesting differences between *Giardia* and other cells. For instance, while AcPh is a soluble enzyme in *Giardia*, it exists as a membrane protein in all cells described so far [81], [82], [83]. The presence of a YXXØ-type internalization sequence in these type-I membrane AcPhs allows several cycles of plasma membrane internalization and recycling for transport to the lysosome. Moreover, while the AcPh tail interacts with AP2 in cells as diverse as *Leishmania* and humans, the lysosomal traffic of *Giardia* AcPh depends on AP1 [13]. Since much of the machinery involved in lysosomal trafficking is derived from a few protein families performing the same basic mechanistic function, the analysis of the similarities and differences between organisms might provide further insight into parasite behavior and eukaryotic cell evolution.

## Supporting Information

Figure S1 **V5-tagged AcPh localizes to the PVs, bare zone and ER.** (A) Electromicrograph of a growing *Giardia* trophozoite showing the PVs located underneath the plasma membrane (in red) and the bare area (in blue). Nuclei (*) and flagella (fg) are also shown. Bar, 0.5 µm. (a) Electromicrograph of the control using secondary antibody alone. Bar, 0.2 µm. (B) Enlarged immunoelectromicrograph of the PVs. AcPh-V5 seems to be detected inside the PVs (arrowhead). (C) Enlarged electromicrograph of the bare area showing some AcPh-V5 localization. (D) Immunoelectromicrograph showing the distinctive distribution of AcPh-V5 on the body of the cell. Bar, 0.1 µm.
(TIF)

Table S1 **Table shows AcPh associated proteins analyzed by LC-MS/MS.** SDS-PAGE, and submitted to the Research Technologies Branch for Protein Identification (NIAID, NIH) for analysis. After three independent experiments, three proteins associated with AcPh were identified (Figure 2A–B and Table S1).
(DOC)
