# Supplementary figures and images for: Vacuolar Protein Sorting Receptor in Giardia lamblia
Source: PLoS One. 2012 Aug 20;7(8):e43712. doi: 10.1371/journal.pone.0043712 (PMC3423367; doi:10.1371/journal.pone.0043712)

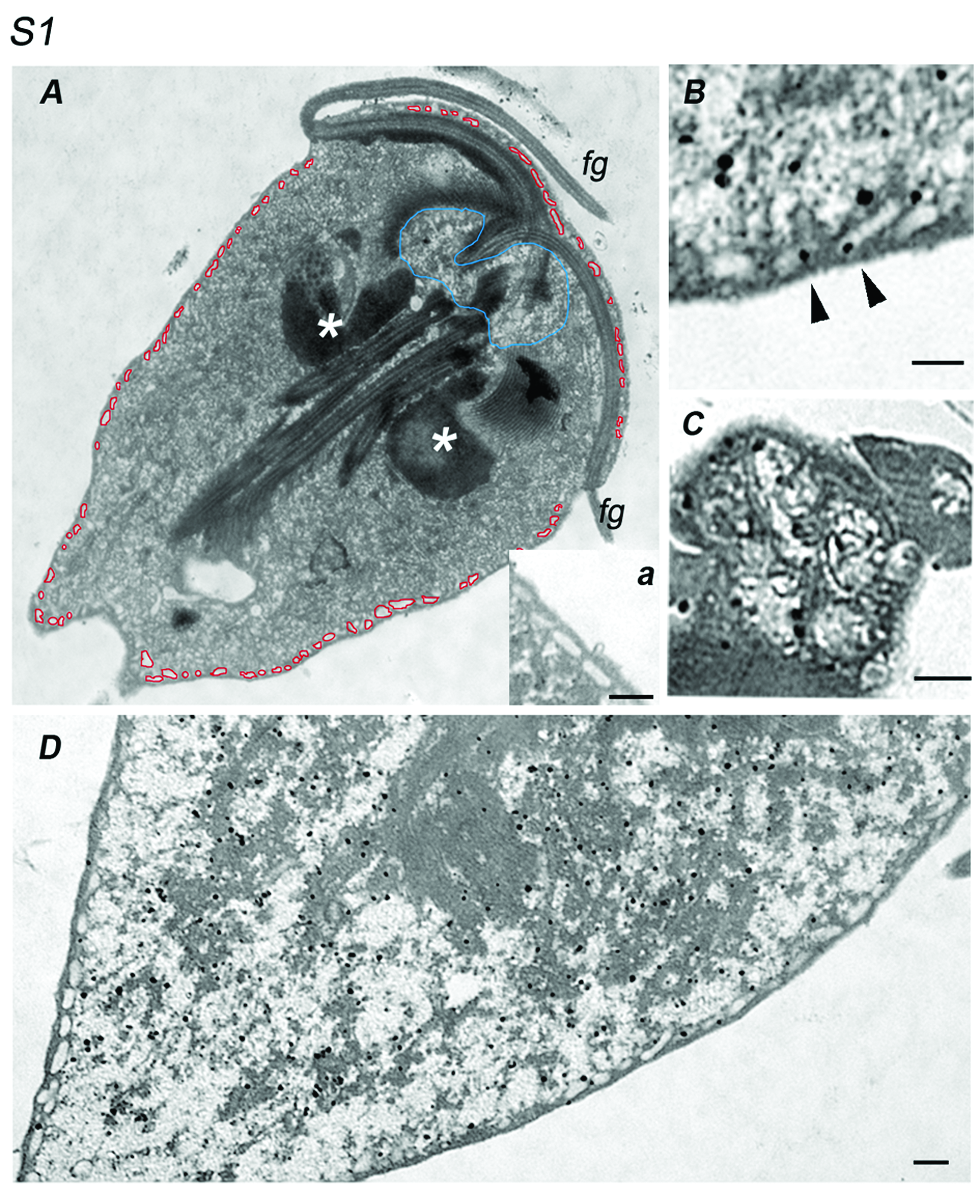

Supplement: Figure S1 — V5-tagged AcPh localizes to the PVs, bare zone and ER. (A) Electromicrograph of a growing Giardia trophozoite showing the PVs located underneath the plasma membrane (in red) and the bare area (in blue). Nuclei (*) and flagella (fg) are also shown. Bar, 0.5 µm. (a) Electromicrograph of the control using secondary antibody alone. Bar, 0.2 µm. (B) Enlarged immunoelectromicrograph of the PVs. AcPh-V5 seems to be detected inside the PVs (arrowhead). (C) Enlarged electromicrograph of the bare area showing some AcPh-V5 localization. (D) Immunoelectromicrograph showing the distinctive distribution of AcPh-V5 on the body of the cell. Bar, 0.1 µm. (TIF) [file pone.0043712.s001.tif]
