# Supplementary material for: Vacuolar Protein Sorting Receptor in Giardia lamblia
Source: PLoS One. 2012 Aug 20;7(8):e43712. doi: 10.1371/journal.pone.0043712 (PMC3423367; doi:10.1371/journal.pone.0043712)
Supplement: Table S1 — Table shows AcPh associated proteins analyzed by LC-MS/MS. SDS-PAGE, and submitted to the Research Technologies Branch for Protein Identification (NIAID, NIH) for analysis. After three independent experiments, three proteins associated with AcPh were identified (Figure 2A–B and Table S1). (DOC) [file pone.0043712.s002.doc]

**Supplementary Table 1:** Results Mass Spectrometry: MS+MS/MS. Research Technologies Branch for Protein Identification (NIAID, NIH).

| **Accession Number** | **Protein Name** | **Species** | **Protein MW** | **Protein PI** | **Pep Count** | **Sequence Coverage** |
| --- | --- | --- | --- | --- | --- | --- |
| GL50803_14070 | kinesin-like protein | *Giardia lamblia*  WB1267 | 87004.1 | 8.84 | 8 | 14 |
| # Assay | Start-End | Mr (expt) | Mr (calc) | Delta | Mis | Sequence |
| 1 | 90-107 | 1878.30 | 1878.01 | -0.01 | 0 | THTMGFEADTPALSEDR |
| 1 | 111-122 | 1398.00 | 1398.61 | 0.04 | 0 | KEEGVCGIFFNR |
| 1 | 229-242 | 1614.12 | 1614.82 | 0.04 | 0 | KQSSQLFITSAEYL |
| 1 | 671-681 | 1510.98 | 1510.80 | 0.01 | 0 | KRTIVPPFSYFR |
| 2 | 543-560 | 2072.85 | 2072.35 | -0.07 | 0 | CSTHVEESLYSLLVNQCF |
| 2 | 707-726 | 2173.00 | 2173.52 | -0.02 | 1 | IFAGKQAEIDFVTAYANMAL |
| 3 | 111-122 | 1398.15 | 1398.61 | 0.03 | 0 | KEEGVCGIFFNR |
| 3 | 401-418 | 1870.93 | 1870.24 | 0.04 | 0 | MVAIVTLSPDLASLPETL |
|  |  |  |  |  |  |  |
| **Accession Number** | **Protein Name** | **Species** | **Protein MW** | **Protein PI** | **Pep Count** | **Sequence Coverage** |
| GL50803_23833 | Vacuolar protein sorting 35 | *Giardia lamblia*  WB1267 | 87504.6 | 6.05 | 8 | 15 |
| # Assay | Start-End | Mr (expt) | Mr (calc) | Delta | Mis | Sequence |
| **1** | 260-270 | 1260.99 | 1260.47 | 0.04 | 0 | IYLSEMISTGF |
| **2** | 143-162 | 2136.12 | 2136.50 | 0.02 | 1 | FLHANPDTAPAELLKLGMPT |
| **2** | 318-337 | 2047.71 | 2047.41 | 0.01 | 0 | KLFVALDDVFAAVVSGCHGV |
| **2** | 653-670 | 2099.13 | 2099.35 | -0.01 | 0 | DDIYNELAAAILVYECFH |
| **3** | 123-138 | 1858.33 | 1858.24 | 0.00 | 0 | LYLMALAASIWLEHLN |
| **3** | 123-140 | 2043.77 | 2043.42 | 0.02 | 0 | LYLMALAASIWLEHLNNA |
| **3** | 564-580 | 1960.81 | 1960.31 | 0.02 | 0 | CIQSFHGALDCYNMLMN |
| **3** | 443-452 | 1442.00 | 1442.55 | -0.04 | 1 | SYAEQVENRHLP |
|  |  |  |  |  |  |  |
| **Accession Number** | **Protein Name** | **Species** | **Protein MW** | **Protein PI** | **Pep Count** | **Sequence Coverage** |
| GL50803_28954 | Hypothetical protein | *Giardia lamblia*  WB1267 | 59183.6 | 7.53 | 8 | 20 |
| # Assay | Start-End | Mr (expt) | Mr (calc) | Delta | Mis | Sequence |
| **1** | 522-536 | 1326.98 | 1326.43 | 0.04 | 0 | CDGVDSACATALGGS |
| **2** | 47-53 | 1332.03 | 1332.59 | -0.04 | 0 | REPTLSLMSWL |
| **2** | 254-283 | 2054.55 | 2054.42 | 0.00 | 0 | LSPITSFVISGQCTAIAMSQ |
| **2** | 342-360 | 2010.03 | 2010.17 | -0.01 | 0 | GGDNVMLGGEDLINELYNS |
| **3** | 522-536 | 1326.33 | 1326.43 | 0.00 | 0 | CDGVDSACATALGGS |
| **3** | 88-104 | 1770.65 | 1770.12 | 0.03 | 0 | LNFTPPPAILVCSDVIA |
| **3** | 507-519 | 1342.11 | 1342.60 | -0.04 | 0 | TFLTGVVLPPLGE |
| **3** | 342-355 | 1403.96 | 1403.53 | 0.03 | 0 | GGDNVMLGGEDLIN |
|  |  |  |  |  |  |  |
| **Accession Number** | **Protein Name** | **Species** | **Protein MW** | **Protein PI** | **Pep Count** | **Sequence Coverage** |
| GL50803_7556 | Acid phosphatase precursor | *Giardia lamblia*  WB1267 | 45657.0 | 5.40 | 8 | 28 |
| # Assay | Start-End | Mr (expt) | Mr (calc) | Delta | Mis | Sequence |
| **1** | 28-45 | 2065.03 | 2065.25 | -0.01 | 0 | RTTLNPFPAETEDWICDG |
| **1** | 49-61 | 1303.88 | 1303.44 | 0.03 | 0 | IAFSTLQAGSTAH |
| **1** | 341-356 | 1796.10 | 1796.16 | 0.00 | 0 | LLYNSKPFVPPFCASI |
| **2** | 113-128 | 2024.58 | 2024.22 | 0.02 | 2 | FVQEAFYIRSTNYERT |
| **2** | 292-309 | 1900.64 | 1900.27 | 0.02 | 0 | LLLYSAHDTTLAPLMGAL |
| **3** | 206-218 | 1362.17 | 1362.55 | -0.03 | 0 | LAGFNETVGWGVL |
| **3** | 341-353 | 1524.98 | 1524.85 | 0.00 | 0 | LLYNSKPFVPPFC |
| **3** | 341-359 | 2067.00 | 2067.48 | -0.02 | 0 | LLYNSKPFVPPFCASITIG |

Protein ID report contains the proteins that have been identified positively by their score in the Mascot database search. # Assays: number of independent assays (1 to 3). Measured peptide mass (Mr expet). Theoretical mass of the peptide (Mr calc). Delta: difference in Mascot score between the best and second best peptide sequence match. Mis: number of missed cleavage sites by tripsin. Sequence: Sequence of the identified peptide. Sequence Coverage by the identified peptides is expressed in %.
